# Multi-scale error-driven dense residual network for image super-resolution reconstruction

**DOI:** 10.1371/journal.pone.0330615

**Published:** 2025-09-18

**Authors:** Xueri Li, Lei Yang, Shimin Liang, Jianfang Wu

**Affiliations:** 1 School of Computer Science, Guangdong University of Science and Technology, Dongguan, China; 2 Faculty of Data Science, City University of Macau, Macau, China; 3 College of Big Data and Internet, Shenzhen Technology University, Shenzhen, China; Khalifa University, UNITED ARAB EMIRATES

## Abstract

Image super-resolution reconstructs high-resolution images from low-resolution inputs. However, current single-image super-resolution techniques often struggle to capture multi-scale information and extract high-frequency details, which compromises reconstruction quality. Moreover, the prevalent feed-forward network architectures lack robust feedback mechanisms for iterative refinement and enhanced acquisition of high-frequency information. To overcome these limitations, this research develops advanced strategies for multi-scale feature extraction, fusion, and feedback in single-image super-resolution. We propose an innovative error-driven, multi-scale dense residual network (EMDN) that retains a feed-forward structure while integrating error-driven feedback. Specifically, our approach utilizes dual multi-scale features: one derived from convolutional kernels of varying sizes and another extracted from diverse inputs, both processed concurrently. Comparative evaluations across different scaling factors demonstrate that our method outperforms existing approaches in both subjective and objective assessments. In particular, compared to the baseline feed-forward network, our model achieves improvements of up to 0.385% in peak signal-to-noise ratio and 0.191% in structural similarity index measure. The experimental results validate the effectiveness and practical significance of our proposed method in enhancing image resolution and restoration quality.

## Introduction

The most important information humans obtain from the outside world is visual, and a key carrier of visual information is images. In early information age, due to device limitations and insufficient transmission rates, acquired images often lacked high resolution and contained less information. With rapid development of communication technology and computers, people can use more high-resolution images, and quality requirements have increased. Advances in high-resolution imaging technologies have enabled cameras and sensors to capture more detailed image content. As the demand for higher image quality has grown, super-resolution techniques have been used to enhance image resolution. In combination with advances in communications and computer technology, these techniques can now be applied more broadly to improve image quality. High-resolution images offer enhanced visual effects, information and details. High-resolution images are in high demand in many areas of production. Specifically, in remote sensing images, more localized information is needed to probe the ground, in medical images, high-resolution images with more details of body tissues are needed to assist doctors in medical diagnosis [1]. Acquiring high-resolution medical images typically necessitates the use of expensive imaging equipment. Moreover, increasing scanning frequency or extending scanning duration to achieve higher resolution can introduce motion artifacts and elevate the physical risks to patients. In contrast, employing super-resolution reconstruction provides a more cost-effective and lower-risk approach to obtaining high-resolution medical images [2–4]. High-precision super-resolution reconstruction of facial images offers substantial potential in law enforcement for personnel identification and screening [5]. However, optical degradations—stemming from factors such as lens blur and aperture diffraction—can significantly compromise the detailed information present in these images [6,7]. In various computer vision applications, utilizing high-resolution images has proven beneficial for achieving superior outcomes. For instance, in target detection, acquiring high-resolution images facilitates identifying smaller targets [5,8,9]. As such, enhancing image resolution stands as a vital area of inquiry [10].

The high resolution of an image means having more pixel points per unit of space. Having more pixel points also means that the image can contain more visual information and can reproduce the real situation of the scene more realistically [11,12]. Despite efforts to optimize the acquisition, transmission, and processing of images, external factors beyond our control may result in varying degrees of quality degradation. Although higher-quality imaging equipment can often improve image resolution, this solution may not be feasible for certain situations due to associated costs and other insurmountable challenges, such as historical images that cannot be reacquired and environments where better equipment cannot be deployed [32]. Therefore, improving hardware alone may not be a sufficient solution to address image resolution challenges. To improve image quality, resorting to software algorithms for image resolution reconstruction is necessary [13]. The process of image super-resolution is not contingent on acquiring superior image-capturing equipment and is cost-effective and widely applicable.

Since the concept of image super-resolution was introduced, numerous approaches have been pro-posed in this field. Traditional super-resolution algorithms can be categorized into three types: interpolation-based methods, reconstruction-based methods, and learning-based methods. However, traditional approaches suffer from insufficient detail restoration, reliance on unrealistic prior assumptions, and low computational efficiency [35,36]. These inherent constraints have catalyzed the emergence of deep learning paradigms in super-resolution, whereby self-learning systems deploy data-driven mechanisms to establish sophisticated nonlinear mappings between low- and high-resolution domains. This technological evolution has considerably enhanced both the perceptual fidelity and functional applicability of super-resolution methods. Modern neural architectures exhibit marked improvements in reconstruction quality relative to conventional algorithms, primarily due to their ability to (1) reduce reliance on human-engineered constraints by autonomously optimizing feature representations, and (2) uncover latent patterns in large datasets that surpass human perceptual capabilities. These data-derived insights facilitate the preservation of photorealistic textures and semantically consistent structures during upscaling operations, representing a paradigm shift in computer vision methodologies [35,37]. Deep learning methods are a hot research topic in image super-resolution. Despite achieving excellent results, current deep learning-based methods have challenges including ineffective multi-scale feature fusion, lack of feedback adjustment information, and slow running speed due to a large model with many parameters. We propose an error-driven and multi-scale based dense residual network (EMDN) to address these issues.

(1) Super-resolution algorithms often rely on a single type of multi-scale features, but a new module has been designed to extract multi-scale features using both multi-scale inputs and multi-scale convolution kernels.

(2) Combined with an error-driven, multi-scale dense residual network, our method efficiently re-constructs image super-resolution. The network leverages two different multi-scale features: one for extracting multi-scale features using various convolutional kernel sizes, and another for multi-scale features of different sizes.

(3) The proposed network excels at various mag-nification ratios, delivering superior results in both subjective and objective assessments compared to other methods.

The remainder of this paper is organized as follows. In Section Related works, we provide a systematic review of super-resolution reconstruction research. Section Materials and methods details our proposed methodology and model architecture. Comprehensive experimental evaluations and ablation studies are presented in Section Experiments and analysis. Finally, Section Conclusion concludes the paper and outlines directions for future research.

## Related works

Many image super-resolution algorithms have been developed. Single-image super-resolution reconstruction presents a significant challenge [14]. Deep learning has dramatically improved super-resolution reconstruction, turning it into a thriving research area [15]. The structure of deep learning-based super-resolution reconstruction is usually designed as an end-to-end approach to generate features, discover mapping connections, and construct high-resolution images automatically.

Chen et al. [16] put forward a model named SRCNN, which was a significant advancement in the field. Its architecture contains three convolutional layers. The process begins by extracting features from a low-resolution image using convolution, which establishes mapping relationships. Compared to conventional super-resolution techniques, SRCNN, based on convolutional neural networks, has superior reconstruction quality. Later, Dong et al. proposed FSRCNN, adding transpose convolution to the original method. Unlike SRCNN, FSRCNN inputs a low-resolution image of the original size, not an interpolated one. Then, transpose convolution reconstructs the high-resolution image, significantly improving reconstruction speed. However, enlarged images using transpose convolution tend to have a checker-board effect on the reconstructed high-resolution image. Wang et al. [17] proposed ESPCN, which re-organizes individual pixel points from multiple low-resolution feature channels into a single unit of a single high-resolution image feature channel - an operation called pixel shuffle. This avoids the checkerboard artifacts of transpose convolution and maintains fast reconstruction speed.

Since He proposed ResNet, the residual structure has been widely used, and adding residual connections can further deepen networks and enhance feature extraction [18–23]. Kim et al. [18] used residual connections for super-resolution in VDSR, with glob-al residual connections mitigating gradient vanishing and enabling deeper networks that significantly improve reconstruction. Kim et al. [24] used RNN structure for super-resolution in DRCN, benefiting from parameter sharing and fewer model parameters. These methods demonstrate satisfactory lower-magnification reconstruction, but struggle at higher magnifications. Li et al. [25] proposed LapSRN, using a stepwise amplification approach and a pyramid-shaped network structure. This reduces complexity and enables better high-magnification results. Lim et al. [21] removed batch normalization (BN) from their EDSR model to further expand network size and achieve excellent reconstruction, although over-sized models are computationally expensive and harder to train. Fang et al. [26] adapted the traditional iterative back-projection method to deep learning with DBPN, using up-projection and down-projection modules for scaling and self-correction. DBPN achieves strong reconstruction results.

In recent years, significant progress has been made in super-resolution technology through advancements in model architecture optimization, degradation modeling, and cross-domain applications. The diffusion model-based MRKD [33] addresses the issues of poor detail consistency and slow sampling speed in traditional DDPM by incorporating multimodal constraints and knowledge distillation. The lightweight hybrid architecture ESRT [34] combines CNN’s local feature extraction with Transformer’s global modeling capabilities, reducing computational costs while maintaining performance advantages. For few-shot scenarios, DESRGAN [35] employs dual-stream feature extraction and artifact suppression loss functions to mitigate overfitting caused by insufficient data.

In degradation modeling and perceptual optimization, the frequency-domain loss function based on the DCT domain [39] significantly enhances the visual quality of reconstructed images by weighting high-frequency information through quantization matrices. The RSISR survey [36] systematically summarizes challenges in real-world degradation modeling and proposes domain adaptation and self-learning methods to bridge the gap between synthetic and real data. SSIR [37] innovatively introduces spatial shuffle multi-head self-attention (SS-MSA), achieving efficient global-local feature fusion while reducing parameters by 40% and improving reconstruction accuracy.

Cross-domain technology migration provides new insights for SISR: the dual-stream convolution and attention modules in the hyperspectral detection framework HCD-Net [40] could inspire multimodal super-resolution designs. The global-local contrast optimization strategy from the medical imaging enhancement algorithm G-CLAHE [42] may improve super-resolution preprocessing quality. The ensemble learning model in COVID-19 diagnosis [41] demonstrates the robustness of multi-model collaboration, offering references for joint optimization of complex degradations in super-resolution tasks. Additionally, the dynamic resource allocation concept from supply chain AI research [38] could be adapted for the lightweight deployment of super-resolution models.

Super-resolution networks utilize feedforward techniques to map low-resolution to high-resolution images. The goal of this work is to build upon this research and provide solutions to challenges outlined in the previous research. Specifically, we introduce a novel method merging error-driven and multi-scale strategies for notable outcomes in image super-resolution reconstruction.

## Materials and methods

### Multi-scale feature extraction block

**Fig 1:**
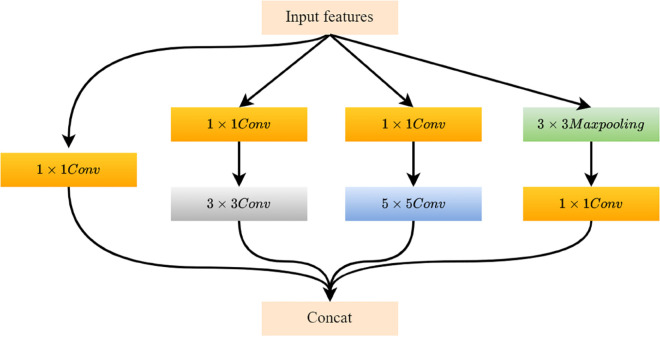
Multi-scale feature extraction block.

Multi-scale features, interpreted as signal sampling at varying granularities, allow observations of different features at different scales. Fig 1 shows their ex-traction process using convolution kernels at multiple scales and fusing the resultant multi-scale features.

Multi-scale feature extraction is achieved through the use of convolutional kernels of varying scales. The approach involves utilizing two separate branches. While larger kernel sizes are capable of obtaining a broader perceptual field, they also entail a higher number of parameters which can negatively impact the network’s operational speed.

### Error-driven fusion block

**Fig 2:**
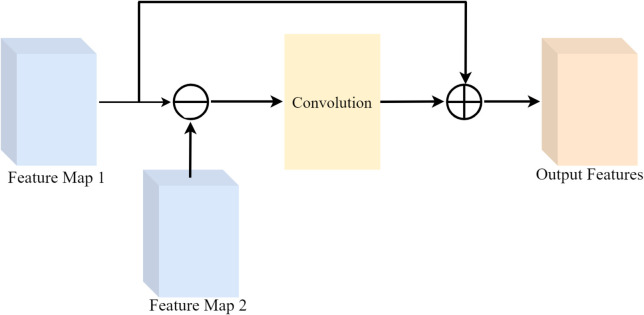
Error-driven structure.

The error-driven approach, as featured in DBPN [27], eliminates the need for a cyclic structure to conduct feedback operations. It employs a feedforward operation to fuse feedback information with the original features, using a process that involves subtracting two feature maps, extracting error information via convolution, and adding the result to the original feature map. In the DBPN framework, up-projection and down-projection modules utilize this methodology to determine reconstruction errors and extract features. The present study leverages these concepts to formulate an error-driven mechanism for feature fusion. In contrast to the prior error-driven approach, the proposed method does not alter the feature map size and is better suited for parallel multi-branch and serial multi-scale feature fusion. The error-driven mechanism visually represented in Fig 2 can be mathematically described by the given Eq (1):

$$\begin{array}{c} F=C_{3\times 3}(F_{1}-F_{2})\\ F_{out}=F+F_{1} \end{array}$$ (1)

Where $C_{3\times 3}$ is the convolution kernel size of 3×3 convolution layer, *F*_1_ and *F*_2_ are the two feature maps, and *F*_*out*_ represents the fused features.

### The overall structure

**Fig 3:**
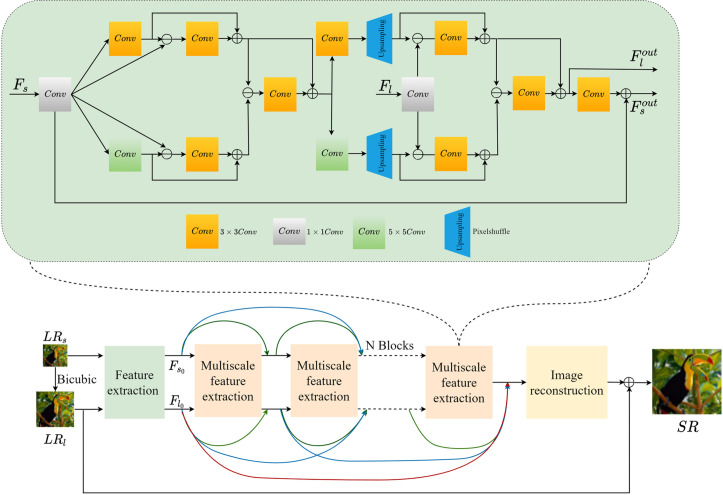
Overall structure of the error-driven and multi-scale based dense residual network. The whole structure contains a preliminary feature extraction module, an error-driven and multi-scale feature extraction module, and an image reconstruction module.

Three components make up the suggested error-driven and multi-scale based dense residual network, as seen in Fig 3: an image reconstruction module, an error-driven and multi-scale feature extraction module, and a preliminary feature extraction module. An error-driven, multi-scale feature extraction module receives the low-resolution image. Dense residual connections are used to extract multi-scale information and transfer them to the subsequent module. The reconstruction module creates the high-resolution image by using the output features from the preceding module.

The low-resolution image’s single-scale features must be extracted before going into the error-driven, multi-scale feature extraction module. There are two types of input low-resolution images: the original size and an interpolated and enlarged version. Both are initially passed through two convolutional layers in order to extract preliminary features. The multi-scale feature extraction module that follows thereafter receives these first features. This module is specifically designed to further extract multi-scale features. The complete procedure is mathematically formulated in Eq (2):

$$\begin{array}{c} F_{S_{0}}=C_{3\times 3}^{S_{2}}(C_{3\times 3}^{S_{1}}(LR_{s}))\\ F_{L_{0}}=C_{3\times 3}^{L_{2}}(C_{3\times 3}^{L_{1}}(LR_{l})) \end{array}$$ (2)

where $C_{3\times 3}$ is the convolution kernel size of 3×3 convolution layer, $F_{S_{0}}$ is the output feature map of original size, and $F_{L_{0}}$ is the output feature map of large size.

The incoming feature map is first subjected to a dimensionality reduction operation, using a bottle-neck layer to compress the feature map to a smaller dimension. The proposed neural network architecture employs a 1×1 convolutional kernel in its bottleneck layer, as mathematically formulated in Eq (3):

$$\begin{array}{c} F_{S}=C_{1\times 1}^{s}([F_{S_{0}},F_{S_{1}},\cdots ,F_{S_{i-1}}])\\ F_{L}=C_{1\times 1}^{l}([F_{L_{0}},F_{L_{1}},\cdots ,F_{L_{i-1}}]) \end{array}$$ (3)

$\left[F_{S_{0}},F_{S_{1}},\cdots ,F_{S_{i-1}}\right]$ is the feature fusion operation. The compressed original size features enter the convolution layer with a convolution kernel of 3×3 and the convolution layer with a convolution kernel of 5×5, respectively. The process in question can be succinctly expressed by the following Eq (4), which provides a clear and concise representation of the underlying mathematics involved.

$$\begin{array}{c} F_{S_{2}}^{1}=C_{3\times 3}(F_{S})\\ F_{S_{2}}^{2}=C_{5\times 5}(F_{S}) \end{array}$$ (4)

$F_{S_{2}}^{1},F_{S_{2}}^{2}$ are the extracted features.

The two features, meticulously extracted from the input data, are fused with the compressed, shallow features separately. The resulting feature maps are then subjected to a subtraction operation, yielding a comprehensive comparison of the two feature sets. This subtraction process allows for the identification and extraction of key error information. The result-ant error information is subsequently added back to the original feature map. The entire procedure is succinctly represented by Eqs (5)–(6).

$$\begin{array}{c} \begin{array}{c} F_{1}=C_{3\times 3}(F_{S_{2}}^{1}-F_{S})\\ F_{S_{3}}^{1}=F_{S_{2}}^{1}+F_{1} \end{array} \end{array}$$ (5)

$$\begin{array}{c} F_{2}=C_{3\times 3}(F_{S_{2}}^{2}-F_{S})\\ F_{S_{3}}^{2}=F_{S_{2}}^{2}+F_{2} \end{array}$$ (6)

Unlike previous methods, here instead of using residual connections to achieve feature fusion, error-driven is used to fuse features. After fusing the shallow feature information, the feature fusion of two branches is performed once using the error-driven, and the information between the two features is ex-changed through the fusion, the feature maps of the two paths are subtracted, the error information is extracted using the convolutional layer, and the error information is returned to the original features, the process described above is expressed by Eq (7) as follows:

$$\begin{array}{c} F_{3}=C_{3\times 3}(F_{S_{3}}^{1}-F_{S_{3}}^{2})\\ F_{S_{4}}^{1}=F_{S_{3}}^{1}+F_{3} \end{array}$$ (7)

After exchanging information, the fused feature map continues to extract features once in the parallel path using convolutional layers with different size convolutional kernels, and after extraction an up-sampling operation is performed on the features, andThe up-sampling multiplier corresponds to the scaling multiplier, and this process is described by Eqs (8)–(9).

$$\begin{array}{c} F_{S_{5}}^{1}=C_{3\times 3}(F_{S_{4}}^{1})\\ F_{L_{1}}^{1}=U_{Pixe}^{1}(F_{S_{5}}^{1}) \end{array}$$ (8)

$$\begin{array}{c} F_{S_{5}}^{2}=C_{3\times 3}(F_{S_{4}}^{1})\\ F_{L_{1}}^{2}=U_{Pixe}^{2}(F_{S_{5}}^{2}) \end{array}$$ (9)

$U_{Pixe}^{1},U_{Pixe}^{2}$ are upsampling operations, which utilize the method PixelShuffle [22]. The principal function of PixelShuffle, a sophisticated technique for pixel reorganization, is to take a feature map that has a relatively low resolution and manipulate it in such a way that it results in a feature map with a significant-ly higher resolution. The process by which this trans-formation occurs involves a combination of convolution and reorganization, which takes place between multiple channels within the image data.

The fusion of the two enlarged features with the input compressed large-size feature, denoted as *F*_*L*_, is performed using the same error-driven fusion method previously applied to the original-size features. This fusion process is mathematically expressed in Eqs (10) and (11):

$$\begin{array}{c} F_{4}=C_{3\times 3}(F_{L_{1}}^{1}-F_{L})\\ F_{L_{2}}^{1}=F_{L_{1}}^{1}+F_{4} \end{array}$$ (10)

$$\begin{array}{c} F_{5}=C_{3\times 3}(F_{L_{1}}^{2}-F_{L})\\ F_{L_{2}}^{2}=F_{L_{1}}^{2}+F_{5} \end{array}$$ (11)

After fusing the shallow, large-scale features, the parallel features are integrated. Specifically, the two large-scale features are combined into a single representation using an error-driven approach, as formulated in Eq (12).

$$\begin{array}{c} F_{6}=C_{3\times 3}(F_{L_{2}}^{1}-F_{L_{2}}^{2})\\ F_{L}^{out}=F_{6}+F_{L_{2}}^{1} \end{array}$$ (12)

where $F_{L}^{out}$ denotes the large-scale characterization of the model, which is used for the reconstruction of the final high-resolution image and as a shallow feature to be passed into the deeper modules. The utilization of the interpolated low-resolution images, along with the subsequent features generated from them, necessitates a considerable amount of computational resources. This is due to the intricate nature of the image processing and analysis involved. In order to make efficient use of these resources and reduce the overall consumption of the entire model, the features can be down-sampled to their original size. This process results in a feature map that is suit-able for multi-scale feature extraction. The mathematical representation of this down-sampling process can be expressed through the following Eq (13).

$$F_{S}^{out}=C_{3\times 3}(F_{L}^{out})+F_{S}$$ (13)

The output features are concatenated with the features outputted by the previous module and fed to the next in a dense residual concatenation. The reconstruction module input is the output of the er-ror-driven multi-scale feature extraction module and the preliminary feature extraction module. The high-resolution reconstruction is performed by means of Eq (14):

$$SR=C_{3\times 3}([F_{L_{0}},F_{L_{1}}^{out},\cdots ,F_{L_{n}}^{out}])+Y_{0}$$ (14)

where *SR* denotes the final generated high-resolution image, *Y*_0_ denotes the global residual connection.

## Experiments and analysis

### The experiment setup and results analysis

This work was performed on Ubuntu 20.04, based on PyTorch 1.8.1, with an NVIDIA GeForce RTX 3090 GPU. The initial learning rate was set to 0.0001, and the number of iterations was set to 300. The learning rate was reduced by half after 200 iterations, using the Adam optimizer. The batch size was set to 16, and the loss function was the mean squared error loss between the reconstructed image and the real image.

This study, which seeks to provide a comprehensive analysis of image data, strategically utilizes the initial 800 images from the DIV2K dataset as the primary training set. The study further selects five separate test sets: Set5 [23], Set14 [24], BSDS100, Manga109 and Urban100. These datasets collectively constitute the foundational evaluation framework for image super-resolution by encompassing a diverse range of scene types (natural, urban, and artistic), challenge dimensions (including fine details, edges, and stylistic elements), and evaluation requirements (from rapid testing to rigorous validation). Their significance stems not only from the consistency of their task objectives but also from their complementary nature, which enables a comprehensive assessment of algorithm performance [43,44]. During the training phase with DIV2K, the images were divided into size patches and trained on RGB color space. The low-resolution images were obtained from their high-resolution counterparts through the application of bicubic degradation, a process commonly used to reduce the resolution of an image. We take peak signal-to-noise ratio (PSNR) and structural similarity (SSIM) as evaluation metrics. These metrics, PSNR and SSIM, are especially well-suited for comparing the degree of similarity between two images. PSNR and SSIM rely exclusively on simple mathematical operations (e.g., mean squared error and local pixel statistics) without requiring complex models or specialized hardware support, rendering them particularly suitable for rapid validation in large-scale experiments. By achieving an optimal balance between efficiency, versatility, and interpretability, these metrics remain an indispensable standard in the field of super-resolution [45–47]. Moreover, the current study made use of reference-free image evaluation metrics, specifically Spatial-Spectral Entropy-based Quality (SSEQ) and Natural Image Quality Evaluator (NIQE), as tools to appraise the quality of super-resolved images in authentic, real-world settings. This approach is adopted because traditional metrics such as PSNR and SSIM, which require an ideal high-resolution reference, are not feasible when such references are unavailable. Therefore, SSEQ and NIQE offer a robust alternative for evaluating the quality of super-resolved images derived from authentic, real-world data [48,49].

The present study involves a comparative analysis of the algorithms proposed herein with those put forth by other researchers, taking into account two objective metrics, namely, PSNR and SSIM. the algo-rithms compared are Bicubic [14], SRCNN [15], VDSR [18], LapSRN [20], MSRN [22], EDSR [21], MDCN [25], SeaNet [26], HDRN [27] and HBPN [28], all comparisons are tested in the mentioned test sets, and the magnifications for comparison are 2 ×, 3 ×, 4 ×, and 8 ×, specifically the image is first downsampled at the corresponding magnification and then restored using a super-resolution algorithm at the same magnification, and the reconstructed quality is evaluated by calculating the PSNR and SSIM by comparing the reconstructed image with the original image. Among them, the error-driven and multi-scale based dense residual network in this pa-per contains 14 multi-scale feature extraction mod-ules. The comparison results are shown in Table 1, Table 2, Table 3 and Table 4.

**Table 1:** Quantitative comparison of different image super-resolution algorithms at 2× magnification.

Algorithm	Scale	Set5	Set14	BSDS100	Urban100	Manga109
		PSNR/SSIM	PSNR/SSIM	PSNR/SSIM	PSNR/SSIM	PSNR/SSIM
Bicubic [14]	×2	33.66 / 0.923	30.24 / 0.869	29.56 / 0.843	26.88 / 0.840	30.80 / 0.934
SRCNN [15]	×2	36.66 / 0.954	32.45 / 0.907	31.36 / 0.888	29.50 / 0.895	35.60 / 0.966
VDSR [18]	×2	37.53 / 0.959	33.05 / 0.913	31.90 / 0.896	30.77 / 0.914	37.16 / 0.974
LapSRN [20]	×2	37.52 / 0.959	32.99 / 0.912	31.80 / 0.895	30.41 / 0.910	37.53 / 0.974
MSRN [22]	×2	38.07 / 0.961	33.68 / 0.918	32.22 / 0.900	32.32 / 0.930	38.64 / 0.977
EDSR [21]	×2	38.11 / 0.960	33.92 / 0.920	32.32 / 0.901	32.93 / 0.935	39.10 / 0.977
MDCN [25]	×2	38.19 / 0.961	33.86 / 0.920	32.32 / 0.901	32.92 / 0.936	39.09 / 0.978
HDRN [27]	×2	37.75 / 0.959	33.49 / 0.915	32.03 / 0.898	31.87 / 0.925	38.07 / 0.977
HBPN [28]	×2	38.13 / 0.961	33.78 / 0.921	32.33 / 0.902	33.12 / 0.938	39.30 / 0.979
SeaNet [26]	×2	38.08 / 0.961	33.75 / 0.919	32.27 / 0.901	32.50 / 0.932	38.76 / 0.977
EMDN (ours)	×2	38.24 / 0.961	33.83 / 0.922	32.36 / 0.902	32.80 / 0.934	39.55 / 0.980

**Table 2:** Quantitative comparison of different image super-resolution algorithms at 3× magnification.

Algorithm	Scale	Set5	Set14	BSDS100	Urban100	Manga109
		PSNR/SSIM	PSNR/SSIM	PSNR/SSIM	PSNR/SSIM	PSNR/SSIM
Bicubic [14]	×3	30.39 / 0.868	27.55 / 0.774	27.21 / 0.739	24.46 / 0.745	26.95 / 0.856
SRCNN [15]	×3	32.75 / 0.909	29.30 / 0.822	28.41 / 0.786	26.24 / 0.799	30.48 / 0.912
VDSR [18]	×3	33.67 / 0.921	29.78 / 0.832	28.83 / 0.799	27.14 / 0.829	32.01 / 0.934
LapSRN [20]	×3	33.82 / 0.923	29.87 / 0.832	28.82 / 0.798	27.07 / 0.828	32.21 / 0.935
MSRN [22]	×3	34.48 / 0.928	30.40 / 0.844	29.13 / 0.806	28.31 / 0.856	33.56 / 0.945
EDSR [21]	×3	34.65 / 0.928	30.52 / 0.846	29.25 / 0.809	28.80 / 0.865	34.17 / 0.948
MDCN [25]	×3	34.69 / 0.929	30.54 / 0.847	29.26 / 0.810	28.83 / 0.866	34.17 / 0.949
HDRN [27]	×3	34.24 / 0.924	30.23 / 0.840	28.96 / 0.804	27.93 / 0.849	33.17 / 0.942
SeaNet [26]	×3	34.55 / 0.928	30.42 / 0.844	29.17 / 0.807	28.50 / 0.859	33.73 / 0.946
EMDN (ours)	×3	34.73 / 0.930	30.68 / 0.853	29.26 / 0.810	28.83 / 0.866	34.17 / 0.949

**Table 3:** Quantitative comparison of different image super-resolution algorithms at 4× magnification.

Algorithm	Scale	Set5	Set14	BSDS100	Urban100	Manga109
		PSNR/SSIM	PSNR/SSIM	PSNR/SSIM	PSNR/SSIM	PSNR/SSIM
Bicubic [14]	×4	28.42 / 0.810	26.00 / 0.703	25.96 / 0.668	23.14 / 0.658	24.89 / 0.787
SRCNN [15]	×4	30.48 / 0.863	27.50 / 0.751	26.90 / 0.710	24.52 / 0.722	27.58 / 0.856
VDSR [18]	×4	31.35 / 0.883	28.02 / 0.768	27.29 / 0.727	25.18 / 0.754	28.83 / 0.887
LapSRN [20]	×4	31.54 / 0.885	28.19 / 0.772	27.32 / 0.727	25.21 / 0.756	29.09 / 0.890
MSRN [22]	×4	32.25 / 0.896	28.63 / 0.783	27.61 / 0.738	26.22 / 0.791	30.57 / 0.910
EDSR [21]	×4	32.46 / 0.897	28.80 / 0.788	27.71 / 0.742	26.64 / 0.803	31.02 / 0.914
MDCN [25]	×4	32.48 / 0.899	28.83 / 0.788	27.74 / 0.742	26.69 / 0.805	31.10 / 0.916
HDRN [27]	×4	32.23 / 0.896	28.58 / 0.781	27.53 / 0.737	26.09 / 0.787	30.43 / 0.908
HBPN [28]	×4	32.55 / 0.900	28.67 / 0.785	27.77 / 0.743	27.30 / 0.818	31.57 / 0.920
SeaNet [26]	×4	32.33 / 0.897	28.72 / 0.786	27.65 / 0.739	26.32 / 0.794	30.74 / 0.913
EMDN (ours)	×4	32.59 / 0.900	28.93 / 0.796	27.76 / 0.742	26.63 / 0.801	31.27 / 0.919

**Table 4:** Quantitative comparison of different image super-resolution algorithms at 8× magnification.

Algorithm	Scale	Set5	Set14	BSDS100	Urban100	Manga109
		PSNR/SSIM	PSNR/SSIM	PSNR/SSIM	PSNR/SSIM	PSNR/SSIM
Bicubic [14]	×8	24.39 / 0.657	23.19 / 0.568	23.67 / 0.547	21.24 / 0.516	21.68 / 0.647
SRCNN [15]	×8	25.33 / 0.689	23.85 / 0.593	24.13 / 0.565	21.29 / 0.543	22.37 / 0.682
VDSR [18]	×8	25.93 / 0.724	24.26 / 0.614	24.49 / 0.583	21.70 / 0.571	23.16 / 0.725
LapSRN [20]	×8	26.15 / 0.738	24.35 / 0.620	24.54 / 0.586	21.81 / 0.581	23.39 / 0.735
MSRN [22]	×8	26.59 / 0.725	24.88 / 0.596	24.70 / 0.541	22.37 / 0.598	24.28 / 0.752
EDSR [21]	×8	26.97 / 0.775	24.94 / 0.640	24.80 / 0.596	22.47 / 0.620	24.58 / 0.778
HBPN [28]	×8	27.17 / 0.785	24.96 / 0.642	24.93 / 0.602	23.04 / 0.647	25.24 / 0.802
HDRN [25]	×8	27.09 / 0.768	24.76 / 0.638	24.68 / 0.598	22.36 / 0.616	24.40 / 0.778
EMDN (ours)	×8	27.26 / 0.787	25.32 / 0.664	24.95 / 0.603	22.84 / 0.634	25.13 / 0.799

In contrast to traditional methods such as Bicubic, the utilization of deep learning techniques confers significant benefits. Furthermore, the proposed method exhibits marked advantages when com-pared to other deep learning methods. Notably, disparities in reconstruction efficacy between the proposed approach and other methods are not apparent when zooming in by a factor of two. This is due to the relatively low difficulty of the 2× zooming task, which fails to provide a clear reflection of differences between various methods. However, the proposed EMDN achieves optimal results across all datasets when zooming in by a factor of three. In addition, EMDN exhibits optimal reconstruction effects on two datasets when zooming in by a factor of four, suboptimal and near-optimal results on two datasets, and optimal results on three datasets when zooming in by a factor of eight.

In our experiments, low-resolution images with different magnifications are obtained by downsampling, and different downsampling magnifications result in different degrees of image information loss. The larger the downsampling magnification, the more image information is lost. During downsampling, more information is lost as the magnification increases. 2× downsampling may only lose a small amount of high-frequency details, while 8× downsampling may lose a large amount of details, making it difficult for the super-resolution algorithm to recover all the original information, corresponding to the results in Tables 1–4.

**Fig 4:**
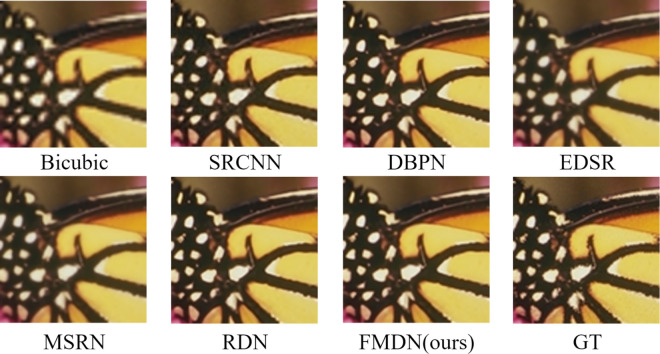
Reconstruction results of each method on the Set14 dataset at a magnification of 2×.

The present study displays the visual effect plots of butterfly images magnified 2 times on the Set5 dataset, implemented by various models, as present-ed in Fig 4. In terms of subjective assessments, the compared methods encompass Bicubic, SRCNN, DBPN, EDSR, MSRN, RDN, and the newly-proposed EMDN, where GT signifies the initial high-resolution image.

**Fig 5:**
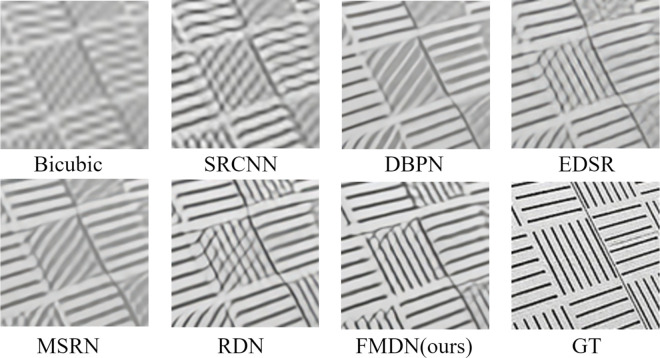
Reconstruction effect of each method on Urban100 dataset at 4× magnification.

The study presents a comparison of various super-resolution techniques for enhancing building images in Urban100. Specifically, the comparison was conducted for the magnification 4× case, which is known to be more challenging than the magnification 2×. The results of the comparison, as depicted in Fig 5, indicate that while images reconstructed through Bicubic and SRCNN appear blurred, those generated through other super-resolution methods display obvious distortion in their patterns. In contrast, the proposed technique is shown to have a high degree of similarity with the original image, without any significant distortion of its shape. A closer examination of the reconstructed results of other methods reveals the presence of large-scale non-existent slashes, errors in line direction, and inadequate restoration of straight lines. The proposed method, on the other hand, stands out from previous approaches by skirting these limitations and exhibiting superior capability in reconstructing high-resolution images with richer, more intricate details that are markedly closer to the original.

Concurrently, in instances where authentic images lack associated high-resolution counterparts, it becomes imperative to employ reference-free methods of assessing image quality. In this research, two reference-free metrics of image quality evaluation, namely NIQE and SSQE, were utilized for comparative analysis. The Bicubic, SRCNN, RDN, DBPN, and EDSR methods were incorporated in the comparison, and their outcomes were presented in Table 5. The method presented in this paper can achieve good results in the reconstruction of real images.

**Table 5:** Comparison of NIQE and SSEQ indicators at 4× magnification.

Metrics	Bicubic	SRCNN	RDN	DBPN	EDSR	EMDN (ours)
NIQE	13.38	13.02	12.74	11.29	11.88	11.20
SSEQ	51.3237	46.8516	49.9867	46.4601	46.9292	46.4190

The model introduced in the paper is carefully evaluated and rigorously compared with several other prevalent methods, with a particular focus on the computational cost. We take the running time as an evaluation metric. The compared methods include SRCNN, VDSR, DRRN, SAN, RDN, and DBPN, and the comparison results are shown in Table 6.

**Table 6:** Comparison of individual models on the Set5 dataset.

Algorithm	Parameter	Multi&Adds	Times(ms)
SRCNN [16]	0.06M	52.5G	2
VDSR [18]	0.7M	612.6G	8
DRRN [29]	0.3M	6796.9G	66
SAN [30]	15.7M	2890.0G	128
RDN [31]	22.6M	1300.7G	91
DBPN [27]	10M	5715.4G	28
EMDN (ours)	17M	490.4G	67

Compared to earlier image super-resolution algorithms, recent methods for parameter count, computation, and runtime have increased. However, the reconstructed images are clearer. Compared to current outstanding methods, this article proposes a method that increases fewer parameters but greatly reduces computation, achieving comparable or even better results.

The presented model is compared with other methods in terms of computational cost. The comparison includes the number of parameters and multiple accumulation operations (Multi&Adds) of the model at 4× magnification, using images of the same size when comparing the number of multiple accumulation operations (Multi&Adds). Finally, the running time is also compared, with input images of size 64×64 and output images of size 256×256. The compared methods include SRCNN, VDSR, DRRN, SAN, RDN, and DBPN, Table 6 shows the comparison of the models on the Set5 dataset.

Compared to earlier image super-resolution algorithms, recent methods for parameter count, computation, and runtime have increased. However, the reconstructed images are clearer. Compared to current outstanding methods, this article proposes a method that increases fewer parameters but greatly reduces computation, achieving comparable or even better results.

### Ablation analyses

To evaluate the effectiveness of the error feedback mechanism, we replace error feedback fusion in the model with traditional cascade fusion. The transformed network is called a multiscale feature dense residual network (MDN). Comparisons are made at 4× magnification, and the degraded network has the same number of layers as the EMDN, ensuring roughly equivalent parameter counts. Comparison results are shown in Table 7. Error feedback feature fusion outperforms traditional cascade fusion across three datasets. In comparison to MDN, our EMDN consistently improves SSIM by over 0.1%, thereby demonstrating that error feedback feature fusion significantly enhances image reconstruction quality.

**Table 7:** Comparison of feedback fusion and cascade fusion.

Algorithm	Set5	Set14	BSDS100
MDN	32.09/0.8935	28.23/0.7814	27.57/0.7357
EMDN	32.17${\;\;}_{+0.25\%}$/0.8945${\;\;}_{+0.11\%}$	28.25${\;\;}_{+0.07\%}$/0.7825${\;\;}_{+0.14\%}$	27.60${\;\;}_{+0.11\%}$/0.7368${\;\;}_{+0.15\%}$

Next, we explore the role of serial multiscale features in the network, which can be obtained by removing dense residual connections from the original network to create a feedback multiscale network (FMN). Since there are two types of serial multiscale feature transfer in the network, one for small feature maps and one for large feature maps, due to the design of three different networks. FMN-s removes dense residual connections for small feature maps, FMN-l removes them for large feature maps, and FMN-non removes all residual connections. In the 4× magnification, for comparison, all above networks have the same size, and the comparison results are shown in Table 8.

**Table 8:** Comparing EMDN with networks that remove serial multi-scale feature connection.

Algorithm	BCDS100	Enhancement	Urban100	Enhancement	Manga109	Enhancement
FMN-non	27.57 / 0.7361	-	25.99 / 0.7854	-	30.44 / 0.9086	-
FMN-l	27.58 / 0.7364	0.036%/0.041%	26.02 / 0.7855	0.115%/0.013%	30.53 / 0.9092	0.296%/0.066%
FMN-s	27.59 / 0.7363	0.073%/-0.027%	26.07 / 0.7861	0.308%/0.089%	30.46 / 0.9089	0.066%/0.033%
EMDN	27.60 / 0.7368	0.109%/0.095%	26.09 / 0.7869	0.385%/0.191%	30.54 / 0.9100	0.329%/0.154%

Table 8 indicates that the proposed EMDN outperforms all competing methods across every dataset and metric. Notably, it achieves the most significant improvements on the Urban100 dataset for PSNR (+0.385%) and for SSIM (+0.191%). Ablation studies further demonstrate that removing any component from the serial jump-connected feature fusion architecture degrades the overall reconstruction capability. In particular, the complete exclusion of dense residual connections results in the most substantial decline in image quality, thereby substantiating the critical role of the proposed multi-scale feature fusion framework based on dense residual connections.

The proposed method is experimentally demonstrated to achieve excellent reconstruction results, and ablation experiments verify the effectiveness of the designed structure.

### Generalization evaluation

We collected CT images from The Cancer Imaging Archive (TCIA), a publicly accessible repository hosting a large number of CT scans. Our study utilized three datasets from TCIA: TCGA-CODA (colon adenocarcinoma CT images), TCGA-STAD (stomach adenocarcinoma CT images), and TCGA-ESCA (esophageal carcinoma CT images). From these datasets, 600 CT images were selected to form the training set, with each dataset contributing one-third of the total training data (i.e., 200 images per dataset). Additionally, 20 images per dataset were combined, shuffled, and partitioned into three independent test sets, each comprising 20 CT images. Following the experimental protocols described previously, these images were downsampled by factors of 2×, 4×, and 8× to evaluate the generalizability of the proposed method.

**Fig 6:**
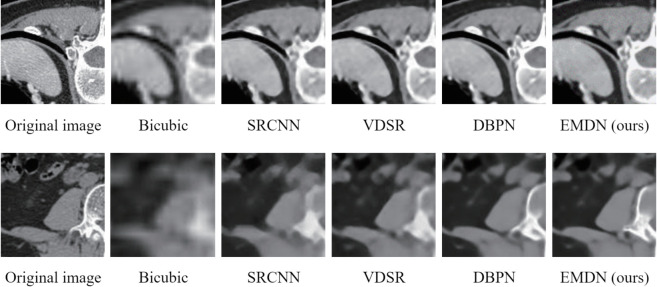
Subjective comparison of various super-resolution reconstruction methods, with the first row displaying 4× upscaling and the second row showing 8× upscaling.

We evaluate our method by comparing it with several alternative approaches on a custom-constructed test set. In our study, the techniques under comparison included Bicubic interpolation, SRCNN, VDSR, and DBPN. The reconstruction quality of CT images was quantified at 2×, 4×, and 8× upscaling factors using objective metrics, namely PSNR and SSIM. As demonstrated in Table 9, deep learning–based methods exhibited significant advantages over traditional approaches, such as Bicubic interpolation, while our proposed method further outperformed the other deep learning techniques. In addition to the quantitative evaluation, we conducted a subjective assessment by visually examining the CT images reconstructed by our method at 4× and 8× magnification levels (see Fig 6). The visual comparisons confirm that our approach produces CT images with a richer representation of details compared to the alternative methods.

**Table 9:** Objective comparison of various super-resolution algorithms across different scaling factors.

test sets	Scale	Bicubic [14]	SRCNN [15]	VDSR [18]	DBPN [27]	EMDN (ours)
		PSNR/SSIM	PSNR/SSIM	PSNR/SSIM	PSNR/SSIM	PSNR/SSIM
Test Set 1	×2	29.22/0.8653	36.68/0.9520	37.53/0.9532	38.36/0.9560	38.47/0.9567
	×4	25.71/0.7806	31.09/0.8761	32.51/0.8852	33.85/0.8950	33.91/0.8954
	×8	22.91/0.7046	25.98/0.7919	28.28/0.8230	29.48/0.8448	29.64/0.8457
Test Set 2	×2	30.24/0.8829	36.82/0.9524	37.69/0.9543	38.85/0.9580	38.95/0.9584
	×4	26.59/0.8106	31.02/0.8862	32.08/0.8954	33.84/0.9079	33.89/0.9084
	×8	23.67/0.7475	26.31/0.8155	28.15/0.8400	29.52/0.8628	29.64/0.8638
Test Set 3	×2	34.10/0.9330	41.08/0.9763	41.91/0.9772	43.13/0.9798	43.24/0.9802
	×4	30.23/0.8843	34.43/0.9377	35.63/0.9445	37.12/0.9525	37.18/0.9529
	×8	26.97/0.8378	29.29/0.8837	30.72/0.9024	31.89/0.9165	31.92/0.9166

The proposed Error-driven Multi-scale Dense Residual Network (EMDN) substantially improves detail recovery in image super-resolution reconstruction by integrating multi-scale feature extraction with an error feedback mechanism. Experimental results demonstrate that EMDN outperforms state-of-the-art approaches (e.g., EDSR, RDN) in PSNR and SSIM metrics for magnification tasks ranging from 2× to 8×, notably reducing artifacts in complex texture reconstruction while maintaining high efficiency with 17 million parameters and 490.4G multiply-add operations. In practical applications, EMDN can be seamlessly integrated into medical imaging systems to enhance CT/MRI resolution for detecting subtle lesions, embedded in surveillance devices to improve low-light face recognition accuracy, and deployed on satellite remote sensing platforms to optimize environmental monitoring. For system deployment, scenario-specific input preprocessing (e.g., bicubic downsampling alignment) and TensorRT acceleration—which achieves real-time processing from 64×64 to 256×256 in 67 ms—are recommended. Future research may involve compressing the model via knowledge distillation for mobile deployment or integrating it with denoising modules to construct end-to-end enhancement pipelines, thereby extending its applicability to autonomous driving and cultural heritage restoration.

## Conclusion

This study introduces an error-driven, multi-scale dense residual network designed to address the prevalent limitation of inadequate high-frequency details in super-resolution images. The proposed single-image super-resolution network strategically integrates error-driven and multi-scale feature fusion by leveraging both the original low-resolution image, which contains essential base information, and its interpolated counterpart, which provides supplementary detail. Experimental results, obtained through rigorous testing, attest to the superior reconstruction performance of the proposed method relative to its contemporaries. Comparative evaluations across various scaling factors indicate that our approach outperforms existing methods in both subjective and objective assessments. Notably, compared to a baseline feed-forward network, our model achieves improvements of up to 0.385% in peak signal-to-noise ratio and 0.191% in structural similarity index measure. Future research will explore the integration of predictive coding techniques into image super-resolution, a promising strategy that may further enhance the accuracy and detail of super-resolved images and push the boundaries of this rapidly evolving field.

## Supporting information

S1 Dataset (DOCX)
