# Supplementary material for: Multi-scale error-driven dense residual network for image super-resolution reconstruction
Source: PLoS One. 2025 Sep 18;20(9):e0330615. doi: 10.1371/journal.pone.0330615 (PMC12445547; doi:10.1371/journal.pone.0330615)
Supplement: S1 Dataset — (DOCX) [file pone.0330615.s001.docx]

Data Availability Statement

The data used in this study are available in the following databases.

DIV2K: https://data.vision.ee.ethz.ch/cvl/DIV2K/.

Set5: https://people.rennes.inria.fr/Aline.Roumy//results/SR\_BMVC12.html.

Set14: https://www.kaggle.com/datasets/ll01dm/set-5-14-super-resolution-dataset.

BSDS100: https://www2.eecs.berkeley.edu/Research/Projects/CS/vision/bsds/BSDS300/html/dataset/images.html.

Urban100: https://www.kaggle.com/datasets/harshraone/urban100.

Manga109: https://www.kaggle.com/datasets/guansuo/manga109.

TCIA: https://www.cancerimagingarchive.net.
